# Computational and Control Methods in Rehabilitation Medicine

**DOI:** 10.1155/2014/707208

**Published:** 2014-07-14

**Authors:** Imre Cikajlo, Takashi Watanabe, Strahinja Dosen

**Affiliations:** ^1^Research and Development Unit, University Rehabilitation Institute, Linhartova 51, 1000 Ljubljana, Slovenia; ^2^Division of Biomedical Engineering for Health and Welfare, Graduate School of Biomedical Engineering, Tohoku University, 6-6 Aoba, Aramaki, Aoba-ku, Sendai 980-8579, Japan; ^3^Department of Neurorehabilitation Engineering, Bernstein Focus Neurotechnology (BFNT) Göttingen, Bernstein Center for Computational Neuroscience (BCCN), University Medical Center Göttingen, University of Göttingen, Von Siebold Street 6, 37075 Göttingen, Germany

In recent years the development of human-machine interfaces for medical applications and rehabilitation medicine has rapidly increased and still continues to grow [1]. The main goals have been a novel in-patient and outpatient diagnostics and improved quality of life, especially for the people with neuromuscular impairments or diseases [2]. The research and development in this field require various novel control algorithms and computational tools to extract features from measured biosignals. Some recently developed algorithms and tools may become part of the in-house equipment for hospitals, while some advanced solutions for home applications may also enable telemedical services in the recent future [3].

Before any computation or control of human activity is possible, information on movement of body segments is required. When information from natural sensors is not accessible, external devices such as accelerometers and gyroscopes can provide sufficient information on gait, standing up, balance, or specific motion of lower or upper extremities. However, these sensors are prone to bias, integration, and temperature drift and cannot provide sufficient accuracy for closed-loop control. The use of various mathematical tools, especially extended Kalman filter, can help to partly overcome the specified problem and can assure measurement of selected parameters up to the acceptable level of accuracy [4]. Besides the Kalman filter alternative feature extraction algorithms like discrete Fourier transform, dynamic time warping, or harmonic linear dynamical system are applied [5]. In this special issue we publish an example of electrocardiogram feature extraction.

The accurate and reliable sensory information is a prerequisite for the design of a closed-loop control system, especially when a human is in interaction with the machine in terms of “feeling the environment” or haptics [6]. The word haptic originates from the Greek word haptikos (*ἅ* *π* *τ* *ι* *κ*ó*ς*) and is related to the sense of touch. This is nowadays particularly important in rehabilitation of patients suffering from stroke. In combination with virtual reality technologies, an upper extremity, and balance, grasping of hand rehabilitation devices can be designed [6]. The haptic robots serving for various rehabilitation purposes are usually complex and intended for the use in clinical environment. However, their simplified passive versions or simple rehabilitation aids equipped with inertial sensory systems are already part of the telerehabilitation systems in function in patients' homes [3] or smart homes. These premises are often well equipped with information-communication technologies and the patients can take full advantage of remote treatment and testing of various novel technologies which may lead to the significant improvement of quality of life [7]. On the other hand, a technological progress, novel computation, and control methods that enable new approaches in rehabilitation medicine require constant clinical evaluation in order to find its way to the everyday clinical practice [8].

We hope the readers of the Journal of Computational and Mathematical Methods in Medicine will find in this special issue solid theoretical and technological solutions as well as their possible applications in the rehabilitation medicine. However, we would like to encourage the readers to raise new research questions and issues which will significantly contribute to the development and rapid uptake of technologies in rehabilitation medicine.

*Imre Cikajlo* *Imre Cikajlo* *Takashi Watanabe* *Takashi Watanabe* *Strahinja Dosen* *Strahinja Dosen*
